# The power and the promise of epigenetic drugs in oncology

**DOI:** 10.3389/fgene.2025.1622115

**Published:** 2026-01-02

**Authors:** Thalita Basso Scandolara, Isabelle Vasconcelos Menegoy Siqueira, Luis Felipe Ribeiro Pinto, Sheila Coelho Soares-Lima

**Affiliations:** 1 Laboratory of Experimental Oncology, Post-Graduation Program in Translational Medicine, Drug Research and Development Center (NPDM), Federal University of Ceará, Fortaleza, Ceará, Brazil; 2 Molecular Carcinogenesis Program, Brazilian National Cancer Institute (INCA), Rio de Janeiro, Brazil; 3 Division of Clinical Research and Technological Development, Brazilian National Cancer Institute (INCA), Rio de Janeiro, Brazil

**Keywords:** cancer, epidrugs, epigenetic modification, oncology treatment, therapy

## Abstract

Epigenetic dysregulation is commonly observed in cancer and has been shown to contribute to different steps of carcinogenesis, from initiation, making cells more prone to transformation, to progression and treatment resistance. Therefore, based on their frequency, relevance and reversibility, epigenetic alterations are promising therapeutic targets in oncology. Although the concept of targeting epigenetic mechanisms is not new, recent advances have been made in reducing toxicity, augmenting specificity, diversifying the targets and combining therapies. In this scoping review, we introduce the main epigenetic mechanisms of gene expression regulation and bring the current knowledge on available epidrugs, focusing on their mechanisms of action and ongoing clinical trials.

## Graphical Abstract

**Figure:**
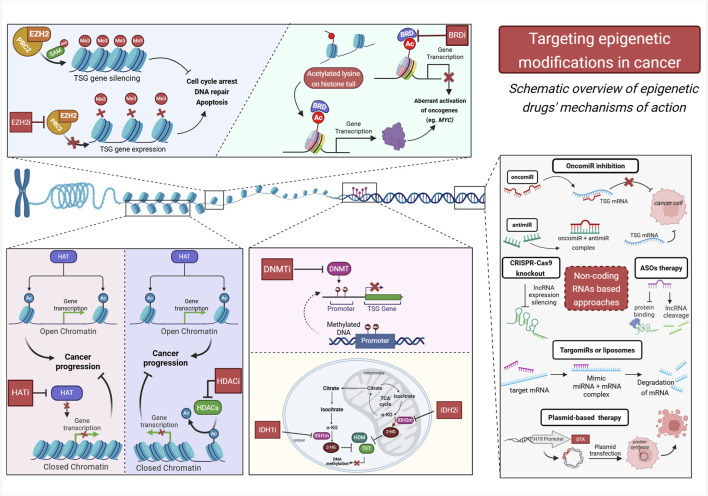
Epigenetic drugs targeting the dysregulated epigenome in cancer may play a crucial role in restoring normal cellular function and provide an alternative approach to counteract these alterations. Currently, the classes of available epigenetic drugs approved by FDA include inhibitors of DNA-methyltransferases (DNMT), the enzymes responsible for DNA methylation; inhibitors of “writers” and “erasers” involved in post-translational modifications of arginines/lysines in histones’ tails, including histone acetyltransferases (HAT) and histone deacetylases (HDAC); inhibitors of histone readers (i.e., bromodomain and extra-terminal domain (BRD) family proteins), which specifically interact with acetylated lysine residues on histones; and inhibitors of isocitrate dehydrogenases 1/2 (IDH1/2) metabolic enzymes. Non-coding RNAs (ncRNAs) can affect several post-transcriptional processes, and among them, the miRNAs are the most commonly studied. Several ncRNAs have shown potential as therapeutic targets.

## Highlights

- Epigenetic modifications represent successful therapeutic targets in hematologic malignancies, but challenges in solid tumors remain due to poor bioavailability and high toxicity
- ncRNAs, including lncRNAs and miRNAs, are emerging as promising therapeutic targets in cancer, with roles in gene expression regulation and potential as diagnostic biomarkers
- Combining epigenetic drugs with immunotherapy is promising for cancer treatment

## 1 Introduction

Epigenetic regulation mainly includes three different processes: DNA methylation, histone modifications, and noncoding RNAs. These are key mechanisms to define cell-specific transcriptional programs, and consequently cell phenotypes, without altering the DNA sequence. The silencing and overactivation of cancer-related genes by epigenetic modifications are common and seem to be a central mechanism during carcinogenesis. Although cancer development is the consequence of multiple events, epigenetic changes are reversible and thus may be translated into therapeutic opportunities (Zhao et al., 2021; Du et al., 2015). Several epigenetic-based drugs have been proposed to treat cancer in the last 20 years, although just a few have been approved by the US Food and Drug Administration (FDA) (Table 1).

**TABLE 1:** US-FDA approved epigenetic-based drugs for cancer therapy.

Drug class	Compound name	Class	Year of approval	Status	Cancer type	Brand name
DNMT inhibitors	Azacitidine	DNMT inhibitor	2004	In use	AML; CML; MDS	Vidaza/Onureg
	Decitabine	DNMT inhibitor	2006	In use	AML; CML; MDS	Decitabine
HDACs inhibitors	Vorinostat	Inhibitor of class I, II, IV HDAC	2006	In use	CTCL; PTCL	Zolinza
	Romidepsin	Inhibitor of class I, II HDAC	2009	In use	CTCL	Istodax/Romidepsin
	Belinostat	Inhibitor of class I, II, IV HDAC	2014	In use	PTCL	Beleodaq
	Panobinostat	Inhibitor of class I, II, IV HDAC	2015	Withdrawn in the US*	Multiple Myeloma	Farydak
IDH inhibitors	Enasidenib	IDH2 inhibitor	2017	In use	Relapsed or Refractory AML	Idhifa
	Ivodesinib	IDH1 inhibitor	2018	In use	Relapsed or Refractory AML; Locally advanced or metastatic cholangiocarcinoma	Tibsovo
	Vorasidenib	IDH1/2 inhibitor	2024	In use	Grade 2 Astrocytoma or Oligodendroglioma	Voranigo
	Olutasidenib	IDH1 inhibitor	2022	In use	Relapsed or Refractory AML	Rezlidhia
EZH2 inhibitors	Tazemetostat	EZH2 competitor	2020	In use	Epithelioid Sarcoma; Follicular Lymphoma;	Tazverik

AML, Acute Myeloid Leukemia; CML, Chronic Myeloid Leukemia; CTCL, Cutaneous T-cell Lymphoma; DNMT, DNA Methyltransferase; EZH2, Enhancer of Zeste Homolog 2; HDAC, Histone Deacetylase; IDH, Isocitrate Dehydrogenase; PTCL, Peripheral T-Cell Lymphoma; MDS, Myelodysplastic neoplasms; US, United States; *Withdrawal on March 24, 2022.

Epigenetic therapy is particularly successful for hematological malignancies, but several trials address and show promising results for solid tumors. Here we aimed to draw together key aspects regarding available epigenetic drugs for clinical use and new approaches that might revolutionize cancer treatment. We also summarized the clinical trials based on epigenetic therapies listed on ClinicalTrials.gov of the National Institutes of Health (NIH) (Supplementary Table S1).

## 2 DNA methylation

DNA methylation is probably the most well-known epigenetic mechanism of gene expression regulation and, not coincidentally, it was the first target for the development of epigenetic drugs. It is a seemingly simple reaction in which a methyl group is transferred from S-adenosylmethionine (SAM, the universal cellular methyl donor) to the carbon five of a cytosine followed by a guanine in the same DNA strand (the so-called CpG sites) (Lopomo and Coppedè, 2018). In humans, three DNA methyltransferases (DNMTs) with enzymatic activity are known, playing different roles in DNA methylation homeostasis (Lopomo and Coppedè, 2018). DNMT1 is usually the enzyme with the highest expression, and it is crucial for the maintenance of cell identity due to its role of copying the methylation patterns to the newly synthesized DNA strand during DNA replication (Hermann et al., 2004; Goto et al., 1994). Besides, DNMT1 also plays a role in the reestablishment of methylation patterns during DNA repair (Mortuse et al., 2005). While DNMT1 is considered a maintenance DNMT, DNMT3A and DNMT3B are described as *de novo* methyltransferases. These enzymes do not require a hemi-methylated template, being able to transfer methyl groups to not previously methylated DNA regions (Okano et al., 1999).

DNA methylation has a broad range of effects. In promoter regions, DNA methylation can regulate gene expression both directly, by promoting the inhibition of the binding of transcription factors able to induce mRNA synthesis or by recruiting transcription factors involved in gene silencing, (Moore et al., 2013), and indirectly, by the recruitment of methyl-binding domain proteins and histones modifiers (Moore et al., 2013). The effects of DNA methylation on other genomic regions are less clear. While in enhancers the mechanisms seem to be similar to those described for promoters (Kreibich and Krebs), the methylation of insulators in general prevents the binding of large proteins such as CTCF (CCCTC-Binding Factor) and enables the approximation between regulatory regions and target genes, resulting in higher expression levels (Yang and Corces, 2011). In gene bodies, the effects are more diverse, with DNA methylation showing a direct correlation with gene expression and regulating alternative splicing (Yang et al., 2014). Finally, DNA methylation is also involved in the maintenance of genomic stability by silencing transposable elements (Jansz, 2019; Jaenisch and Bird, 2003; Kim and Costello, 2017; Nishiyama and Nakanishi, 2021).

Although the DNA methylation profile observed in cancer cells, i.e., hypermethylated promoters and a hypomethylated genome (Nishiyama and Nakanishi, 2021), could raise the question whether the inhibition or the induction of the process would be more beneficial, Farber and Diamond provided the first evidence of the most suitable path in 1948 (Farber et al., 1948). Although their report did not involve specific DNA methylation-targeted drugs, it provided data on the use of folic acid agonists and antagonists. Folic acid is one of the necessary, and not endogenously produced, intermediates of the one-carbon metabolism, which produces SAM for all methylation reactions within a cell (Mentch and Locasale, 2016). By treating children diagnosed with acute leukemia with the folic acid conjugates pteroyltriglutamic (teropterin) or pteroyldiglutamic acids (dopterin), it was possible to observe an acceleration of the leukemic process in post-mortem examinations. Although this could represent a new window of opportunity for the potentiation of other treatments targeted to highly replicating cells, such as radiation, the effects of folic acid antagonists were more promising. Out of 16 patients who were given 4-aminopteroylglutamic acid (aminopterin), 10 responded to the therapy, showing satisfactory, but temporary remissions (Farber et al., 1948). In this seminal study, a concern regarding treatment toxicity, which limited its duration, was reported and was shown to be shared with DNA methylation inhibitors later (Uddin and Fandy, 2021).

**FIGURE 1:**
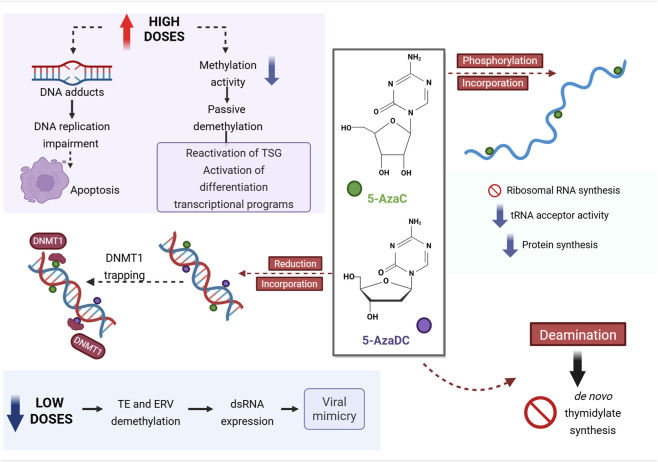
Molecular mechanisms of the hypomethylating agents 5-azacitidine (5-AzaC) and 5-aza-2′-deoxycitidine (5-AzaDC). Upon cellular uptake, 5-AzaC and 5-AzaDC may undergo reduction, phosphorylation or deamination. When reduced, both 5-AzaC and 5-AzaDC are incorporated into DNA during cell cycle. Subsequently, DNA methylation is inhibited, since these analogs are resistant to methyl transfer and DNMT1 becomes trapped. When administered in high doses, 5-AzaC and 5-AzaDC may form DNA adducts, resulting in apoptosis, and/or lead to passive demethylation, which has the potential to reactivate repressed tumor suppressor genes (TSG) and activate differentiation transcriptional programs. 5-AzaC and 5-AzaDC in low doses have been shown to result in the demethylation of transposable elements (TE) and endogenous retroviruses (ERV), inducing the expression of double-strand RNAs (dsRNA), and eliciting viral mimicry. After phosphorylation, 5-AzaC can also be incorporated into RNA molecules, inhibiting ribosomal RNA synthesis and reducing both tRNA acceptor activity and protein synthesis. The deaminated forms of 5-AzaC and 5-AzaDC inhibit *de novo* thymidylate synthesis. DNMT1, DNA methyltransferase 1.

In 1964, a new nucleoside analog was synthesized aiming at inhibiting nucleic acid biosynthesis, 5-azacitidine (5-AzaC) (Pískala and Šorm, 1964). A single dose of 5-AzaC was able to increase survival time in a mouse model of acute leukemia, being referred to as a cancerostatic compound (Šorm et al., 1964). 5-AzaC can incorporate to both RNA (following its phosphorylation) and DNA (following its reduction), presenting more effects than only DNA methylation inhibition (Veselý, 1985) (Figure 1). Indeed, this drug was shown to inhibit ribosomal RNA synthesis, to reduce transporter RNA acceptor activity and protein synthesis (Veselý, 1985; Lee and Karon, 1976). In contrast, 5-aza-2′-deoxycitidine (5-AzaDC) can only incorporate into DNA and it is approximately 10-fold more efficient than 5-AzaC in inducing differentiation (Jones and Taylor, 1980; Li et al., 1970). Within the DNA strand, besides being resistant to methylation, 5-AzaC and 5-AzaDC induce a reduction of DNMT1 activity since these enzymes become irreversibly bound to DNA (Jones and Taylor, 1980; Taylor and Jones; Christman et al., 1983) (Figure 1). The resulting adducts may impair DNA replication during the following cell cycles (Davidson et al., 1992). The ability to bind covalently and consequently inhibit DNMT1 activity makes 5-AzaC and 5-AzaDC toxic effects dependent on DNMT1 levels (Jüttermann et al., 1994; Flatau et al., 1984). Indeed, resistant cells can still incorporate 5-AzaDC to their DNA, but higher DNMT1 levels make it less likely for these enzymes to encounter the cytosine analog (Flatau et al., 1984). Finally, once deaminated into 5-azauridine and 5-aza-2′-deoxyuridine, 5-AzaC and 5-AzaDC, respectively, can also inhibit *de novo* thymidylate synthesis (Veselý et al., 1969), which adds to their toxicity.

Although much less explored, 5-AzaDC inhibitory effects on DNMT3 enzymes have also been suggested (Oka et al., 2005; Suzuki et al., 2006). *Dnmt3*-null mutant embryonic stem cells have been shown to be highly resistant to 5-AzaDC treatment relative to wild-type or single-mutant (*Dnmt3a*, *Dnmt3b* or *Dnmt1*) cells. In addition, the sensitivity to treatment of both undifferentiated and differentiated cells correlated with Dnmt3 expression (Oka et al., 2005). In another study, the repressive effects of Dnmt3 on the activity of a transcription factor was shown to be recovered by 5-AzaDC treatment (Suzuki et al., 2006). These findings indicate that 5-AzaDC mechanisms of action might not be fully elucidated and may depend on specific conditions.

In 2004, 5-AzaC was first approved by the FDA to treat specific subtypes of myelodysplastic neoplasms (MDS). Currently, it is used to treat adults with acute myeloid leukemia who had a first complete remission after intensive induction therapy and who are not able to finish intensive curative therapy; adults with certain types of MDS, including chronic myelomonocytic leukemia (CMML); children aged 1 month and older with newly diagnosed juvenile myelomonocytic leukemia (National Cancer Institute, 2006a). 5-AzaDC (decitabine) was first approved in 2006 for treating adults with MDS, including CMML (National Cancer Institute, 2006b). Although in a much smaller number of studies, in addition to hematological malignancies, these drugs are currently being tested in clinical trials for solid tumors, such as ependymoma, head and neck squamous cell carcinoma, breast cancer, gastric cancer, osteosarcoma, chondrosarcoma, colorectal cancer, melanoma, non-small cell lung cancer, pancreatic adenocarcinoma, mesothelioma, esophageal squamous cell carcinoma, ovarian cancer, prostate carcinoma, gliomas, and *BRCA1/​2*- and Homologous Recombination Deficient (HRD)-Mutated Tumors (clinicaltrials.gov, last accessed on 29 January 2024).

The effectiveness of 5-AzaC and 5-AzaDC against cancer cells is primarily supposed to be based on the cytotoxicity of the adducts formed between these analogs and DNMT1 in the DNA strand. These adducts have been shown to activate the p53-dependent DNA damage response and, therefore, their effects would require functional p53 to induce apoptosis (Karpf et al., 2001). In this context, the much lower frequency of inactivating *TP53* mutations observed in hematological malignancies (10%–20%) relative to solid tumors (>60%) (Peller and Rotter, 2003) could explain, at least in part, the FDA approval and higher number of ongoing clinical trials for those diseases. Additionally, the treatment with demethylating agents can activate differentiation transcriptional programs (Ramakrishnan et al., 2017). This can be especially useful to treat cancers exhibiting low differentiation status. Finally, the reactivation of tumor suppressor genes, often silenced by promoter hypermethylation in cancer, has also been pointed out as one of the anticancer mechanisms of 5-AzaC and 5-AzaDC (Daskalakis et al., 2002).

The severe side-effects associated with 5-AzaC and 5-AzaDC have limited the doses and duration of treatment (Christman, 2002; Yan et al., 2016). Therefore, new approaches focusing on dose reduction and combination with other drugs have been proposed. In this context, almost 10 years after the initial FDA approval, a new anticancer mechanism was described for 5-AzaDC. In 2015, two independent groups have shown that the treatment of cancer cells with 5-AzaDC in low doses does not result in high cytotoxicity but leads to apoptosis *via* an interferon-mediated antiviral response (Roulois et al., 2015; Chiappinelli et al., 2015). This response was called viral mimicry and is the result of double-strand RNA (dsRNA) expression, which elicits the MDA5/MAVS RNA recognition pathway. Interestingly, dsRNA expression was a consequence, at least in part, of the demethylation of endogenous retroviruses (ERVs) and transposable elements. Roulois and colleagues further showed that these effects are more prominent in cancer initiating cells (Roulois et al., 2015), while Chiappinelli and colleagues highlighted that the treatment sensitized to anti-CTLA4 therapy in a pre-clinical melanoma model (Chiappinelli et al., 2015). Later, a clinical trial with solid tumor patients showed that low dose 5-AzaDC treatment induced T-cell proliferation and consequent antitumor response (Li et al., 2017). These findings marked the start of a new road for demethylating agents-based therapy, in which low-doses and combination with immune checkpoint blockade were the “Go” sign.

In parallel, other nucleoside DNMT inhibitors have been developed, focusing on decreasing toxicity and augmenting their half-lives. Guadecitabine is a dinucleotide which consists of decitabine linked by a phosphodiester bond to deoxyguanosine (Daifuku, 2019). Although not yet approved by the FDA, this decitabine prodrug has shown a bigger half-life in the bloodstream due to its resistance to deamination, resulting in reduced infusion times and higher patient convenience (Daifuku, 2019). So far, three Phase 3 clinical trials have been terminated for acute myeloid leukemia, MDS and chronic myelomonocytic leukemia and depicted no superiority relative to the treatment of choice (ClinicalTrials.gov ID: NCT02348489, NCT02907359, NCT02920008). Zebularine, a cytidine analog, is stable both on acidic and neutral pHs, which enables oral administration (Yoo et al., 2004). Although preclinical studies have shown a higher stability and lower toxicity relative to 5-AzaC and 5-AzaDC (Hu et al., 2021), clinical trials are still necessary to prove efficacy.

Despite the current use of 5-AzaC and 5-AzaDC in the clinic and the development of more stable and deamination-resistant nucleoside DNMT inhibitors, their lack of specificity, poor bioavailability, instability, and toxicity are still major concerns for their widespread use in cancer treatment. Based on this, other drugs showing demethylation effects have been repurposed (procainamide and procaine, for example,) and new compounds have been synthesized (Hu et al., 2021). Among the latter, the quinoline-derived compound SGI-1027 is a DNA-competitive and SAM-non-competitive inhibitor of DNMT1 (Gros et al., 2015). Although its mechanisms of action are not completely elucidated, SGI-1027 was shown to reactivate tumor suppressor genes (Sun et al., 2018), and to induce apoptosis and cell cycle arrest in cancer cell lines (She et al., 2020). SGI-1027 analogue MC3353 was able to induce green fluorescent protein expression in demethylating assays and exhibited anti-proliferative effects in different cancer cell models (Zwergel et al., 2019).

Other non-nucleoside inhibitors include RG108, a small molecule (2-(1,3-dioxo-1,3-dihydro-2Hisoindol-2-yl)-3-(1H-indol-3-yl)propanoic acid) selected through an *in silico* screen based on a three-dimensional model of the human DNMT1 catalytic domain. RG108 blocks DNA methylation in cell-free conditions and in human cancer cell lines, leading to the reactivation of epigenetically silenced tumor suppressor genes (Brueckner et al., 2005). Finally, MG98, a second generation antisense oligodeoxynucleotide inhibitor of human DNMT1, was shown to be well-tolerated with early evidence of clinical activity in a Phase I, open-label study, including patients with advanced solid malignancies (Plummer et al., 2009). But showed lack of objective response in a Phase II trial in patients with metastatic renal carcinoma, which might be attributable to a lack of target effect or the choice of tumor type (Winquist et al., 2006). Although still in its infancy, treatment with these new compounds might represent new directions on the road for demethylating agents-based therapy.

## 3 Histone modifications

Genomic DNA is packed by histones, shaping chromatin structure. The dynamic state of the chromatin can be affected by histones’ post-translational modifications, resulting in two main chromatin states. Euchromatin has a more relaxed configuration to facilitate the transcription process, thus this region usually contains highly expressed genes. On the other hand, heterochromatin has a more compact configuration, which is associated with the repression of gene transcription (Yang and Wang, 2021). The precise regulation of chromatin states involves the action of enzymes, known as “writers”, “erasers” and “readers”, responsible for the introduction, removal, and identification of chemical modifications, respectively, in histone tails (Hyun et al., 2017).

Histone modifications can impact DNA configuration, and include methylation, acetylation, phosphorylation, ubiquitylation, and the post-translational addition of other less characterized chemical groups on the N-terminal tails of histones H2A, H2B, H3 and H4 (Zhao and Shilatifard, 2019). Given their central role in the control of cellular processes, these modifications are dynamically added and removed by chromatin-modifying enzymes in a highly regulated and specific manner (Baylin and Jones, 2011).

The enzymes responsible for these modifications play critical roles in the regulation of several key genes for normal cell function (Zhuang et al., 2020; Bannister and Kouzarides, 2011). They include histone acetyltransferases or lysine acetyltransferases (HATs or KATs, respectively), histone deacetylases or lysine deacetylases (HDACs or KDACs, respectively), histone methyltransferases (HMTs, that may act both on lysines, KMTs, and on arginines, PRMTs), histone demethylases (HDM, with specific enzymes responsible for lysine demethylation, KDMs, or arginine demethylation, PRDMs), among others (Barski et al., 2007). The aberrant expression of these enzymes is associated with the disruption of the histone modification machinery, leading to abnormal cellular responses associated with cancer initiation, progression, and metastasis (Baylin and Jones, 2011; Berdasco and Esteller, 2019).

There are a great number of histone modifications found to be involved in cancer development, but acetylation and methylation are the most commonly evaluated ones. They may occur in the same lysine histone residue and can be associated with active or repressed transcription (Yang et al., 2022).

### 3.1 Histone acetylation

Histone acetylation is a fast and reversible process controlled by HATs and HDACs. HATs are the “writers”, as they transfer acetyl groups to lysine residues in histone tails. HDACs are known as “erasers”, since they remove acetyl groups from histone tails (Hyun et al., 2017). HATs can be subdivided into three big families based on their primary sequence homology, similar structural features, and functional roles: GNAT family (Gcn5-related N-acetyltransferases); MYST family (named after the founding members MOZ, Ybf2/Sas3, Sas2, and Tip60) and p300/CBP (protein of 300 kDa and CREB-binding protein) (Huang et al., 2019). The enzymatic acetylation process involves the transfer of an acetyl group from acetyl-coenzyme A to the α/ε-amino group of lysine residues, leading to neutralization of their positive charge and reducing the affinity between histones and DNA (Grunstein, 1997). In general, histone acetylation levels are higher in promoter regions of active genes and can affect both the initiation and elongation of gene transcription (Hyun et al., 2017).

The dysregulation of histone acetylation patterns has been associated with cancer development, as it can be linked to increased oncogene expression (Gil et al., 2017). Several non-histone molecules have also been shown to be acetylated by HATs, such as p53, c-MYC and NF-κB, which can also be associated with the carcinogenic process (Singh et al., 2010; Harachi et al., 2021). In addition, genes that encode HATs can be translocated, amplified, overexpressed and/or mutated in various types of cancer, indicating that HAT inhibitors may represent a potential therapeutic approach for oncological diseases in the future (Hu et al., 2019).

**FIGURE 2:**
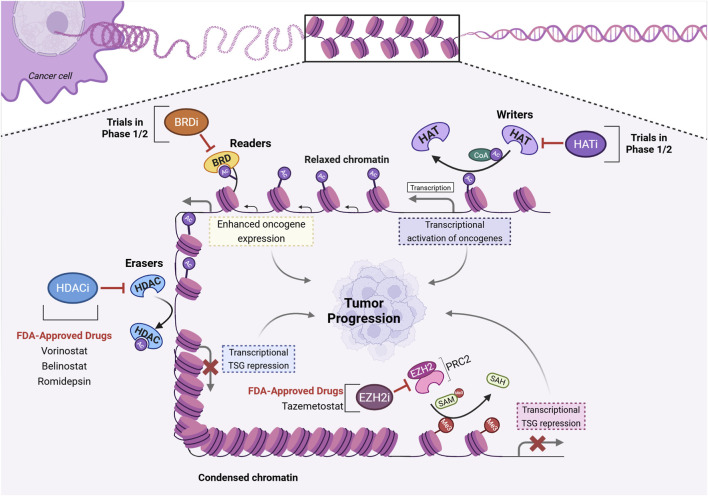
Histone modifications in oncogenesis and current drug-targets. Histones undergo acetylation through the catalytic action of histone acetyltransferases (HAT), also called “writers”. In a general context, the hyperacetylation of histones leads to an open chromatin conformation, facilitating accessibility to transcription factors. HAT inhibitors (HATi) interfere with HAT’s catalytic activity. Bromodomain (BRD)-containing proteins can regulate gene expression by several mechanisms, such as the recognition of acetylated histones (thus being called “readers”), enhancing transcription. Currently available BRD inhibitors (BRDi) are more specific to BET bromo domains, and act to prevent the interaction between the BRD and the acetyl group. Histone deacetylases (HDAC) regulate chromatin remodeling by removing acetyl residues from histone proteins, hence augmenting chromatin condensation and inhibiting gene transcription. Given that HDACs are usually overexpressed in cancer, several inhibitors (HDACi) were developed to interfere with the deacetylation process. As part of the Polycomb Repressive Complex 2 (PRC2), EZH2 catalyzes the trimethylation of histone three at lysine 27 (H3K27me3), which contributes to transcriptional silencing. EZH2 inhibitors (EZH2i) can specifically inhibit the H3K27me3 mark added by PRC2; AcCoa, Acetyl-CoA, Ac, acetyl-groups, SAM, S-adenosylmethionine, SAH, S-Adenosyl-L-homocysteine.

Both natural and synthetic compounds have emerged as promising HAT inhibitors (Figure 2). Among the compounds reported (and recently reviewed elsewhere (Liu et al., 2023), CCS1477 is the most promising candidate, currently in Phase 1/2 clinical trials (De Bono et al., 2019; Brooks et al., 2019; Brooks et al., 2021). During preclinical studies, CCS1477 was capable of inhibiting cell proliferation in a wide range of cancer cell lines, controlling tumor growth more effectively than 5-AzaC (Brooks et al., 2021). CCS1477 is currently in trials to assess safety, tolerability, pharmacokinetics, and biological activity in patients with non-Hodgkin lymphoma, multiple myeloma, acute myeloid leukemia, high-risk myelodysplastic syndrome, metastatic castration-resistant prostate cancer (mCRPC), metastatic breast cancer, non-small cell lung cancer and advanced solid tumors (ClinicalTrials.gov ID: NCT04068597 and NCT03568656).

Garcinol is a natural compound and a potent inhibitor of p300/CBP-mediated acetylation that presents anticancer activity in several *in vitro* and *in vivo* models. However, studies regarding its therapeutic potential are still in early stages, as it requires improvement not only of its activity, but also selectivity and stability (Kopytko et al., 2021). Anacardic acid is derived from medicinal plants and was shown to effectively inhibit Tip60, a HAT member of the MYST family, activity *in vivo* (Sun et al., 2006; Sung et al., 2008); and p300/CBP and PCAF (p300/CBP-associated factor) *in vitro* (Balasubramanyam et al., 2003). Besides, it was also able to sensitize cells to ionizing radiation through the inhibition of Tip60-dependent activation of the ATM pathway, a central mediator required for cells to survive double-strand breaks (Sun et al., 2006; Bernstein et al., 2010). In addition, anacardic acid was shown to induce synthetic lethality in PTEN-deficient cancer cells *in vitro* and *in vivo* (Liu et al., 2020). However, detailed mechanistic studies are still necessary to fully comprehend its potential as a HAT inhibitor, and to enter preclinical and clinical studies.

### 3.2 Histone deacetylation

There are 18 human HDACs grouped according to their yeast orthologs, subcellular location, and catalytic site specificities (Zn^2^+ dependent or NAD-dependent) into four classes: class I (HDACs 1, 2, 3, and 8), which are mostly located in the nucleus; class II, which is further classified into two groups: class IIa (HDACs 4, 5, 7, 9), and class IIb (HDACs 6 and 10), located both in the cytoplasm and nucleus; class III (SIRT1, SIRT2, SIRT3, SIRT4, SIRT5, SIRT6, and SIRT7) located in the cytoplasm, mitochondria, and nucleus; and class IV (HDAC11) located in the plasma membrane, cytoplasm and nucleus (Ho et al., 2020; Li et al., 2020). Histone deacetylation results in compact and supercoiled chromatin, which is associated with transcriptional inhibition (Manal et al., 2016).

Currently, the HDAC inhibitors (HDACi) available and/or in clinical trials (Figure 2) are usually not selective and target the classes I, II, and IV HDACs, but not class III due to their different catalytic mechanism (Witt et al., 2009). HDACi can be classified into four groups, hydroxamates (Vorinostat, Belinostat, Panobinostat), benzamide derivatives (Entinostat, Tucidinostat), cyclic peptides (Romidepsin) and aliphatic acid (Valproic Acid) (Hontecillas-Prieto et al., 2020). Vorinostat (also known as suberoylanilide hydroxamic acid, SAHA) inhibits class I and II HDACs and it was the first HDACi approved by the FDA for the treatment of Cutaneous T-cell Lymphoma (CTCL) in 2006, with approximately 30% of the patients showing clinical benefit (Mann et al., 2007). Despite its initial promise to be beneficial for various tumors, its use as monotherapy is restricted to CTCL patients when other treatment options have failed. Although it has presented almost no benefit as monotherapy (Berdasco and Esteller, 2019; Pan et al., 2016), it is largely tested in combinatorial strategies for a variety of solid tumors (nine ongoing trials on solid tumors–identified in ClinicalTrials.gov, last accessed on 22 February 2024). Mechanistically, SAHA inhibits HDACs by binding to their active sites, resulting in the suppression of genes associated with cell cycle progression and tumor growth, such as cyclin D1, and in the induction of p21 activation (Natarajan et al., 2018). However, it must be noticed that resistance may occur. A recently published study showed that SAHA and Panobinostat induce *NEDD9* expression and promote breast cancer metastasis, which might be one of the reasons for therapeutic failure (Hu et al., 2023).

Belinostat (PXD101), a second-generation analogue of Vorinostat, is a pan-HDAC that inhibits all zinc-dependent HDAC enzymes, with high affinity for class I HDACs 1-3, but also class II HDACs 6, 9, and 10, as well as class IV HDAC (O’Connor et al., 2015). It received accelerated approval in 2014 for the treatment of relapsed or refractory Peripheral T-Cell Lymphoma (PTCL) patients (Lee et al., 2015). A limitation of both Belinostat and Vorinostat is their relatively short half-lives in the bloodstream, due to their rapid metabolic degradation, which negatively affects their effectiveness as drugs (Kenny et al., 2020).

Panobinostat (Farydak) also received accelerated approval in 2015 for the treatment of drug-resistant multiple myeloma in combination with the proteasome inhibitor bortezomib (Sivaraj et al., 2017). However, its approval by FDA was withdrawn in 2022 due to incomplete post marketing clinical trials to verify its clinical benefit (Federal Register Doc. 2022–06182, Docket No. FDA-2022-N-0352). Currently, it is the only HDACi approved by the European Medicines Agency (Tzogani et al., 2018).

Entinostat (MS-275 or SNDX-275) is a synthetic benzamide HDACi, which selectively inhibits class I and IV HDACs. Compared to other HDACi, Entinostat has a prolonged half-life, allowing better dose adjustments (Gore et al., 2008). Entinostat has demonstrated promising antitumor activity in both *in vitro* and *in vivo* cancer models. Preclinical studies have shown promising results for several types of solid tumors when Entinostat was combined with other targeted therapies and chemotherapeutic agents, resulting in enhanced immune activity, drug synergy, and the ability to overcome treatment resistance (Ruiz et al., 2015; Smith et al., 2018; Hicks et al., 2021). However, a recent Phase 3 study (ClinicalTrials.gov ID: NCT02115282) did not report clinical benefit supporting the combination of Exemestane and Entinostat for advanced breast cancer (Connolly et al., 2021). The combination of Entinostat with Olaparib *in vitro* was shown to sensitize homologous recombination-proficient ovarian cancer cells to PARP inhibitors, potentializing the effect of the drug by reducing *BRCA1* expression (Gupta et al., 2021). A Phase 1/2 trial explored this combination, but no results were published yet (ClinicalTrials.gov Identifier: NCT03924245). However, these findings indicate that Entinostat is a promising drug for the treatment of solid tumors, especially breast and ovarian cancer.

Romidepsin (also known as depsipeptide, FK228) is the only natural HDACi approved by the FDA to treat CTCL and PTCL (Coiffier et al., 2012; Piekarz et al., 2009). It was first identified as a compound called FR901228, isolated from the bacteria *Chromobacterium violaceum* (Nakajima et al., 1998), and its active form inhibits class I HDACs, with a slight effect against class II HDAC (VanderMolen et al., 2011). Also, it can induce cell cycle arrest and apoptotic cell death in several solid tumor cells, such as colon cancer (Shi et al., 2021). Thus, Romidepsin might represent a promising treatment option for solid tumors in the next few years.

### 3.3 Histone methylation

Histone methylation is a covalent modification that occurs mainly on lysine and arginine residues using SAM as methyl donor (Greer and Shi, 2012). The effect of histone methylation may vary according to the specific amino acid residue modified (H3K4 and H3K27 methylation have different effects, for example,), number of methyl groups added (mono-, di- or tri-methylated lysines; and mono-, as well as symmetrically or asymmetrically di-methylated arginines), and location of the modified histones within the genome (promoter or gene body, for example,) (Lan and Shi, 2009). Methylation of lysines on H3 and H4, such as H3K4, H3K9, H3K9, H3K27, H3K36, H3K79 and H4K20, are the most well-known histone methylation modifications and some of them are preferentially associated with gene activation or silencing (Barski et al., 2007; Greer and Shi, 2012).

Among HMTs, the Enhancer of Zeste Homolog 2 (EZH2) is one of the most studied enzymes. EZH2 is the catalytic subunit of the Polycomb Repressive Complex 2 (PRC2), which regulates cell development by compacting chromatin and suppressing genes. EZH2 specifically targets H3K27 residues, causing transcription repression through their trimethylation (H3K27me3), as it is a repressive epigenetic mark that favors a closed chromatin state. *EZH2* was firstly found upregulated in prostate cancer and was associated with poor prognosis (Varambally et al., 2002). Now, it is known that both solid and hematologic malignancies can harbor mutations that affect EZH2 activity. There is evidence of its dysregulation in several tumors, such as breast cancer (Kleer et al., 2003), bladder cancer (Wu et al., 2016), pancreatic cancer (Wang et al., 2019), oral cancer (Shih et al., 2017), head and neck cancer (Mochizuki et al., 2018), and others (Kim and Roberts, 2016). Additionally, it has been demonstrated that *EZH2* expression by cancer cells can suppress anti-cancer immune response, diminishing CD8^+^ T cell infiltration in the tumor microenvironment (Peng et al., 2015).

The only EZH2 inhibitor approved by FDA is Tazemetostat (Tazverik) (Figure 2), used for the treatment of metastatic or locally advanced epithelioid sarcoma (Gounder et al., 2020) and follicular lymphoma (Julia and Salles, 2021). Tazemetostat can also inhibit EZH1 activity, which is a homolog of EZH2 within the PRC2 complex with a much lower methyltransferase activity (Margueron et al., 2008). It has been shown to be effective and safe in clinical trials, with tolerable side effects. Thus, it is currently in clinical trials for other types of cancer (Duan et al., 2020).

A few compounds targeting EZH2 are under development and ongoing preclinical or Phase 1/2 clinical trials. So far, studies using *in vivo* models demonstrated growth inhibition in bladder tumors, and when administered to two human patients with a rare and treatment-resistant bladder cancer, almost a complete tumor regression was observed (Adema and Colla, 2022). SHR2554 is a promising EZH2 inhibitor being evaluated for relapsed or refractory mature lymphoid neoplasms and is currently in Phase 1 trial. The first results demonstrated an acceptable safety profile and promising antitumor activity (Song et al., 2022). CPI-1205 is a potent, selective, SAM-competitive EZH2 inhibitor evaluated as a monotherapy for B-Cell Lymphoma. It has also been studied in combination with Ipilimumab for advanced solid tumors, but its trial has been stopped prior to proceeding to Phase 2 (ClinicalTrials.gov ID: NCT03525795). Currently, CPI-1205 is in Phase 1/2 trial for mCRPC, in combination with either enzalutamide or abiraterone/prednisone (Taplin et al., 2019) (ClinicalTrials.gov ID: NCT04068597 and NCT03568656).

### 3.4 Bromodomain inhibitors

A group of domains, named bromodomains (BRDs), enable “reader” proteins to specifically recognize acetylated residues in histone tails (Zeng and Zhou, 2002). The BRD-proteins mainly regulate gene transcription and the recruitment of molecular partners. They are subclassified into two families based on their structure, the Bromodomain and Extra-Terminal Domain (BET) and non-BET families. The BET family is composed by BRD2, BRD3, BRD4, and BRDT, and plays important roles in cancer by directly regulating the expression of cancer-related genes such as *MYC* (Bandopadhayay et al., 2014), and the transcription factor NF-κB (Hajmirza et al., 2018). The aberrant expression of BET proteins promotes oncogenesis by blocking cell differentiation and driving cell growth (Pérez-Salvia and Esteller, 2017). Fourteen clinical trials are ongoing to evaluate the potential of candidate BRD inhibitors, mostly for solid tumors, but also for hematological malignancies (ClinicalTrials.gov, last accessed on 22 February 2024). Nine are in Phase 1, three in Phase 2, and two in Phase 1/2. Although in its infancy, inhibiting BRD-proteins has a significant potential as novel drugs in the field of cancer therapy, as reviewed elsewhere (Pérez-Salvia and Esteller, 2017).

## 4 Mutant isocitrate dehydrogenase-based therapies

Although not initially thought to be directly associated with epigenetics, the tricarboxylic acid cycle enzyme Isocitrate Dehydrogenase (IDH), when mutated in cancer, has been shown to affect different epigenetic mechanisms. Wild-type IDH catalyzes the conversion of isocitrate into α-ketoglutarate (α-KG), with the production of NADPH in the process. Instead, the mutant form of the enzyme is capable of converting α-KG into the so-called oncometabolite D-2-hydroxyglutarate (D-2HG), a reaction that consumes NADPH. Alpha-KG is a cofactor for a number of dioxygenases (α-KG-dependent dioxygenases), including HDMs and TETs (Ten-Eleven Translocation), the latter involved in DNA demethylation. TETs are able to consecutively oxidize 5-meC, 5-hydroxymethylcytosine (5-OHmeC) and 5-carboxylcytosine (5-CaC) into 5-OHmeC, 5-CaC and 5-formylcytosine, respectively. The two last bases are then recognized by the base-excision repair machinery, leading to their replacement by an unmethylated cytosine. Therefore, D-2HG, produced by mutant IDH, competitively inhibits HDMs and TETs, impairing histone and DNA demethylation, respectively, and leads to chromatin restructuring, blockade of cell differentiation and induction of a stem-like transcriptional program, among other effects (Figueroa et al., 2010; Lu et al., 2012; Turcan et al., 2018).

IDH has two isoforms, cytoplasmic IDH1 and mitochondrial IDH2. Both the genes coding these enzymes have been found mutated in cancer, leading to the production of D-2HG, but show different hotspots and affect different tumor types. While the arginine 132 residue (R132) is more commonly affected in IDH1, both the residue arginine 172 (R172, analogous to R132 in IDH1) and arginine 140 (R140) can be mutated in IDH2. *IDH1* mutations are more commonly observed in chondrosarcomas (13.1%), cholangiocarcinomas (51.0%) and gliomas (71.2%); *IDH2* is more commonly affected in angioimmunoblastic T cell lymphoma (25.0%) and sinonasal undifferentiated carcinoma (82.0%); and AML shows a more similar percentage of mutations in the two genes (*IDH1* - 13.1%; *IDH2* - 18.2%) (Pirozzi and Yan, 2021).

**FIGURE 3:**
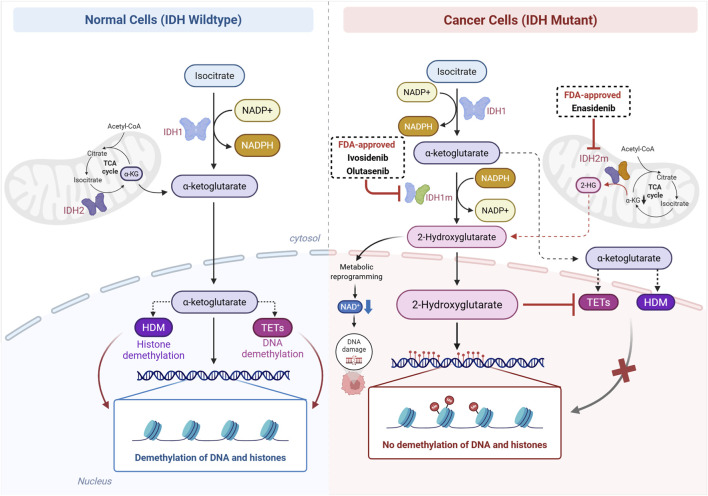
Scheme of wild-type and mutant IDH1/2 molecular functions. Wild-type IDH1/2 enzymes convert isocitrate into α-ketoglutarate, which is necessary for the activity of Ten-Eleven Translocation (TET) enzymes and Histone Demethylases (HDM), promoting demethylation of DNA and histones, respectively. IDH1/2^mut^ enzymes convert the wild-type IDH product, α-ketoglutarate, into the oncometabolite 2-hydroxyglutarate (2-HG) both in the cytosol (IDH1^mut^) and in the mitochondria (IDH2^mut^). In a competitive manner, 2-HG inhibits the activity of TET and HDM enzymes, resulting in a hypermethylation phenotype. Besides, 2-HG is capable of inducing metabolic reprogramming in these cells, further leading to DNA damage. IDH1^mut^i and IDH2^mut^i block this abnormal production of 2-HG, enabling TET and HDM normal function.

Due to the high prevalence of *IDH* mutations in different cancer types and the reversibility potential of the epigenetic alterations induced by D-2HG, inhibitors of the mutant form of the enzyme have been developed (Figure 3). Mutant IDH harbors an unstable regulatory segment, which favors the binding of inhibitors to its allosteric site (Ma and Yun, 2018). In turn, inhibitors prevent the conformational change necessary for catalysis. Mutant IDH1 and IDH2 present different allosteric inhibition pockets (Ma and Yun, 2018), which enabled the development of specific inhibitors. Ivosidenib is an IDH1-mutant-specific inhibitor developed after chemical optimizations of AGI-5198, the first inhibitor developed. It is currently approved by the FDA to treat adult patients with relapsed or refractory AML carrying an *IDH1* mutation as detected by an FDA-approved test (AG-120, Tibsovo; Agios Pharmaceuticals, Inc., MA, USA (Food and Drug Administration, 2018)). The combination of ivosidenib with 5-AzaC further showed a longer event-free survival in *IDH1*-mutant AML patients relative to patients treated with 5-AzaC and placebo (Montesinos et al., 2022). In addition, a Phase 3 clinical trial showed improved progression-free survival in patients with advanced, *IDH1*-mutant cholangiocarcinoma treated with ivosidenib compared with placebo (median 2.7 months [95% CI 1.6–4.2] vs. 1.4 months [1.4–1.6]) (Abou-Alf et al., 2020). Overall survival was also improved, despite a high rate of crossover (Zhu et al., 2021).

Olutasenib is another IDH1-mutant-specific inhibitor recently approved by the FDA to treat adult patients with relapsed or refractory AML. It differs from ivosidenib in chemical structure and binding properties, but it also induces cell differentiation in *IDH1*-mutant primary human AML cells (Venugopal and Watts, 2023). Additionally, it showed better 18-month survival rates (78% vs. 50%) and better median overall survival (11.6 vs. 8.8 months) relative to ivosidenib in their registrational trials (Venugopal and Watts, 2023).

Since AML patients carry mutations in *IDH2* in a similar frequency to *IDH1*, it is not surprising that IDH2-mutant-specific inhibitors are also indicated for its treatment. Enasidenib, approved in 2017 by the FDA, suppresses D-2HG production by both R140 and R172 IDH2 mutants, which precedes but is not predictive of clinical response. Interestingly, mutations in *NRAS* and other MAPK signaling effector genes were enriched in nonresponsive AML patients (Amatangelo et al., 2017), indicating that the mutational profile of these genes should also be evaluated prior treatment indication in addition to *IDH2* mutations. More recently, enasidenib also showed clinical benefit in heavily treated older AML patients carrying *IDH2* mutations in a Phase 3 trial (de Botton et al., 2023).

Vorasidenib, an inhibitor of both mutant *IDH1* and *IDH2*, was tested in a double-blind Phase 3 trial which included 331 patients with residual or recurrent grade 2 *IDH*-mutant glioma (Mellinghoff et al., 2023). The first results showed that progression-free survival was improved by vorasenib relative to placebo (27.7 vs. 11.1 months). The INDIGO trial is currently active, but not recruiting, with an estimated date of completion in 2027 (ClinicalTrials.gov ID: NCT04164901). However, vorasidenib (Voranigo, Servier Pharmaceuticals LLC), has already received FDA approval for adult and pediatric patients (≥12 years) with grade 2 astrocytoma or oligodendroglioma harboring a susceptible *IDH1* or *IDH2* mutation.

In addition to IDH-mutant inhibitors, cancer cells carrying *IDH* mutations have also been shown to be more sensitive to conventional chemotherapy and radiotherapy. as a consequence of metabolic reprogramming associated with the consumption, instead of production, of NADPH by the mutant enzymes (Tateishi et al., 2017; Lu et al., 2017). This reprogramming leads to reduced NAD^+^ levels (the expression of the NAD^+^ salvage pathway enzyme nicotinate phosphoribosyltransferase - NAPRT1 - is downregulated), and to the disturbance of NAD^+^-dependent pathways (Tateishi et al., 2015; Nagashima et al., 2020). Indeed, two independent groups have shown in 2017 that the PARP1-associated DNA repair pathway, highly dependent on NAD^+^ levels, is severely compromised in IDH-mutant cells, making them especially sensitive to PARP inhibitors (Lu et al., 2017; Sulkowski et al., 2017).

Taken together, all the data presented here show quite a promising and fast advance on targeting the vulnerabilities of IDH-mutant cells. As observed for other epigenetic therapies, the major challenge seems to lie on the effectiveness in solid tumors. More specifically, gliomas have an additional hurdle, the blood-brain barrier. Therefore, new research and the development of new drugs should focus on overcoming these limitations.

## 5 RNA-based therapies

**FIGURE 4:**
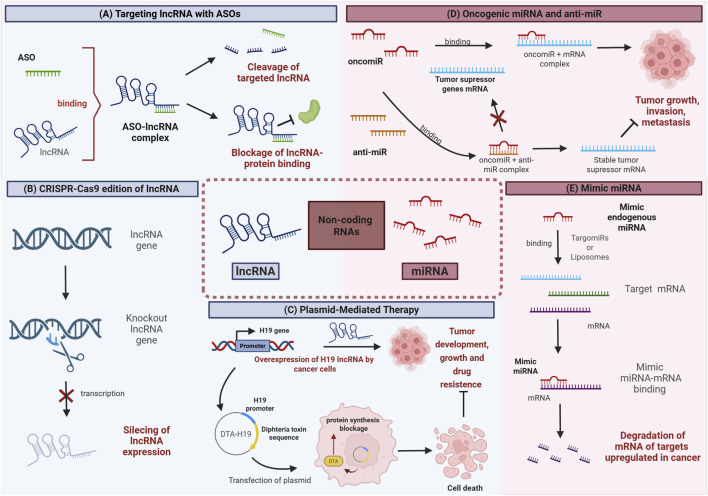
Approaches through which epidrugs can target ncRNAs. **(A)** Antisense oligonucleotides (ASOs) can be designed to bind to complementary sequences within the target lncRNA, leading to its degradation or to the inhibition of its interaction with other molecules. This leads to the disruption of oncogenic lncRNAs function and to the inhibition of cancer cell growth or cell death promotion. **(B)** Application of CRISPR/Cas9 technology to silence the transcription of lncRNAs. **(C)** Mechanism by which the BC-819 plasmid, containing the DTA-H19 construct, contributes to tumor suppression. BC-819 plasmid is introduced into cancer cells, it results in the overexpression of DTA-H19 which selectively targets and kills cancer cells, leading to tumor suppression. **(D)** Inhibition of oncomiRs with synthetic anti-miRs. This mechanism prevents oncomiR binding to target tumor supressor mRNAs, increasing their levels and resulting in the suppression of cancer cell proliferation. **(E)** Synthetic miRNA mimics can be used to target overexpressed mRNAs in cancer cells, leading to their degradation and ultimately inhibiting oncogenic pathways.

Non-coding RNAs (ncRNAs) have emerged as potential targets for cancer therapy, as they have been shown to participate in many stages of carcinogenesis (Winkle et al., 2021). Classified according to their length, ncRNAs can be stratified into long-noncoding RNAs (lncRNA, >200 nucleotides) and small non-coding RNAs, which include microRNAs (miRNAs). They can modulate gene expression by affecting the chromatin structure, transcriptional regulation, and post-transcriptional modifications (Kumar et al., 2020). As an example, they can inhibit the expression of cancer-related genes through RNAi (Miranda et al., 2019; Statello et al., 2021). In this section, we will provide an overview of epigenetic therapeutic approaches, with a particular emphasis on the role of ncRNA-based therapies in the context of cancer treatment (Figure 4).

### 5.1 Long non-coding RNAs

Long non-coding RNAs (lncRNAs) have emerged as pivotal players in the regulation of gene expression and epigenetic processes (Wu et al., 2021). These RNA molecules, typically longer than 200 nucleotides, constitute most non-coding RNAs. Unlike protein-coding RNAs, lncRNAs do not serve as templates for protein synthesis; instead, they play a role in the regulation of different cellular processes, such as chromatin remodeling and transcriptional regulation (Martianov et al., 2007; Wang and Chang, 2011). In recent years, lncRNAs aberrant expression has been observed in several types of cancer, contributing to tumor development and progression (Chi et al., 2019; Peng et al., 2018; Xu et al., 2018). Extensive preclinical research has unveiled the potential of lncRNAs as therapeutic targets in cancer, while clinical trials exploring the application of lncRNAs in therapy are in early stages (Chen et al., 2021).

Different approaches have been used in preclinical studies to target lncRNAs. Antisense oligonucleotides (ASOs) are single-stranded nucleic acid polymers with a length of 15–25 nucleotides. ASOs can be designed to block lncRNA interaction with their binding proteins, or to induce lncRNA’s degradation (Nguyen et al., 2020). In lung and breast cancer, the downregulation of lncRNA-MALAT1 (Metastasis Associated in Lung Adenocarcinoma Transcript 1) with ASOs led to tumor growth inhibition and metastasis (Gutschner et al., 2013; Arun et al., 2016). Similarly, limited tumor formation and metastasis have been observed after ASO-mediated downregulation of lncRNA-*SChLAP1* (SWI/SNF Complex Antagonist Associated With Prostate Cancer 1), which is upregulated in prostate cancer (Huarte, 2015). CRISPR/Cas9 (Clustered Regularly Interspaced Short Palindromic Repeats, CRISPR-Associated Endonuclease 9, Cas9) has also been used to induce the total or partial knockdown of lncRNAs (Li et al., 2023). Zhuo et al. identified lncRNA-GMAN (Gastric Cancer Metastasis Associated) as highly expressed in gastric cancer, and its deletion using the CRISPR/Cas9 system resulted in a reduction of invasiveness and ability to form metastases, improving overall survival in mice (Zibitt et al., 2021; Zhuo et al., 2019).

Another approach for cancer treatment is plasmid-mediated therapy. H19 was one of the first lncRNAs identified and plays a significant role both in embryonic development and tumorigenesis (Smits et al., 2008). In preclinical studies, researchers examined the effectiveness of a plasmid known as BC-819 (DTA-H19), which contains the gene encoding the A subunit of diphtheria toxin under the regulation of the *H19* promoter. DT-A expression is activated by the presence of *H19* transcription factors, which are upregulated in tumor cells (Lu et al., 2018; Smaldone and Davies, 2010). *In vivo* models demonstrated tumor growth inhibition in bladder tumors after administration of BC-819. Furthermore, an almost complete tumor regression was observed in two human patients with a rare and treatment-resistant bladder cancer. Phase 1 and 2 trials evaluated the maximum tolerated dose and the efficacy of BC-819, revealing a favorable safety profile and evidence of anti-tumor activity in at least six out of the 14 patients treated (ClinicalTrials.gov ID: NCT00393809). A similar Phase 2 study is being conducted for other types of cancer such as advanced ovarian cancer, primary peritoneal carcinoma, and unresectable locally advanced pancreatic cancer. Additionally, the safety and efficacy of BC-819 administered with gemcitabine have been evaluated in patients with locally advanced pancreatic adenocarcinoma (ClinicalTrials.gov ID: NCT01413087). None of the above-mentioned clinical trials advanced to Phase 3 so far.

Currently, most clinical trials focusing on lncRNAs in cancer assess their expression levels as potential biomarkers for the detection, prognosis, and complementary diagnosis, including non–small cell lung carcinoma (NSCLC), acute myeloid leukemia (AML), colorectal cancer (CRC), hepatocellular cancer, thyroid cancer, and others (ClinicalTrials.gov, last accessed on 26 February 2024). These trials represent a crucial step toward translating the preclinical promise of lncRNA-targeted epigenetic therapies into clinical practice, potentially opening novel possibilities for cancer treatment.

### 5.2 miRNAs

Approximately 19–24 nt long, miRNAs are another subclass of ncRNAs. Their primary function is to silence specific target genes through the RNA-Induced Silencing Complex (RISC) (Kim et al., 2014). Over the last 2 decades, research has identified miRNAs, known as oncomirs, that are up- or downregulated in cancer. Oncomirs target tumor suppressor genes (Kumar et al., 2020), while other miRNAs act as tumor suppressors by targeting oncogenes (Peng and Croce, 2016).

Three miRNA-based drugs have been approved to advance from Phase 1 to Phase 2 trials for different cancer types (Chakraborty et al., 2021). TargomiRs, more specifically miR-16 mimic, were the first to complete Phase 1 in patients, acting as tumor suppressor in pleural mesothelioma and non-small cell lung cancer (van Zandwijk et al., 2015). This drug also includes a delivery vehicle and an anti-EGFR antibody. Interestingly, miR-16 targets include genes related to cancer progression, such as *CCND1* and *BCL2* (JR, 2018; Pekarsky et al., 2018). *In vivo* studies have shown a well-accepted dosage and early signals of excellent antitumor response in patients. Phase 2 trials plan to include TargomiRs combined with standard chemotherapy (ClinicalTrials.gov ID: NCT02369198) (van Zandwijk et al., 2017; Reid et al., 2013).

MRX34 is a liposomal formulation of miR-34a designed to restore endogenous miR-34a levels. It was first tested in primary liver cancer, small cell lung cancer, lymphoma, multiple myeloma, and renal cell carcinoma (Bader, 2012). This drug exhibited remarkable treatment effectiveness by suppressing multiple oncogenic pathways, including WNT/β-catenin, MAPK, c-MET, Hedgehog, and VEGF, along with genes associated with the p53 pathway (Daige et al., 2014). However, clinical studies lasted only 3 years, as the drug was suspended due to immune-related severe side effects (ClinicalTrials.gov ID: NCT01829971) (Hong et al., 2020).

Cobomarsen (anti-miR-155) is an inhibitor of miR-155, a miRNA upregulated in patients with lymphoma and leukemia, and associated with a poor prognosis (Seto et al., 2018). Both preclinical studies and Phase 1 clinical trials showed miR-155 inhibition and tumor shrinkage, as well as promising results in terms of efficacy, safety, and tolerability (Anastasiadou et al., 2021). Owing to its therapeutic potential and absence of adverse effects, a Phase 2 clinical trial (SOLAR study) investigated Cobomarsen effectiveness in patients with mycosis fungoides subtype of CTCL and disease progression following treatment with Vorinostat, an already approved drug for the treatment of CTCL (Anastasiadou et al., 2021). However, SOLAR study (ClinicalTrials.gov ID: NCT03713320 and NCT03837457) showed lack of superiority relative to Vorinostat and was discontinued (miRagen Therapeutics Inc, 2020).

As reviewed elsewhere, there are several ongoing clinical trials for many types of cancer (i.e., breast, glioma, head and neck, ovarian, and non-small cell lung cancer) evaluating miRNAs potential as diagnostic, therapeutic, and prognostic biomarkers (Reddy, 2015). When searching for the terms “miRNA and cancer” in the clinical trial database, only 12 (out of 42) interventional studies were active or had completed recruitment (ClinicalTrials.gov, last accessed on 22 February 2024). Most of them are focused on the detection of circulating miRNAs as biomarkers to assess treatment response or as outcome indicators, including biomarkers of resistance to neoadjuvant chemotherapy, therapeutic response and hormone sensitivity in breast cancer, and predictive factors for resistance to treatment in metastatic castration-resistant prostate cancer, among others. None of them aimed directly at the development of novel cancer therapies.

## 6 Future directions

Despite the mechanisms and ongoing trials described in this review, the practical future of epigenetic therapy will likely depend on emerging technologies that offer greater selectivity and precision, such as locus-specific epigenome editing using CRISPR systems and targeted protein degradation (PROTACs/Ab-PROTACs). Achieving this goal requires both high specificity and a clear understanding of the mechanistic basis of these platforms.

CRISPR-based epigenome editing, particularly using catalytically inactive dCas9, is a promising strategy to manipulate DNA methylation or demethylation at defined genomic loci, enabling the reactivation of tumor suppressor genes or the silencing of oncogenes without inducing DNA breaks (Urbano et al., 2019). Targeted methylation can be achieved by fusing dCas9 to the catalytic domain of DNMT3A (Vojta et al., 2016), whereas targeted demethylation uses dCas9 fused to the TET hydroxylase catalytic domain (Choudhury et al., 2016). These tools have shown robust activity in preclinical cancer models, including melanoma and colorectal cancer, and represent a shift toward programmable epigenetic manipulation, as highlighted in the recent review by Gupta et al. (Gupta et al., 2025). Nonetheless, several limitations remain, including the need to improve editing efficiency in deep tissues, minimize off-target effects, and develop delivery systems that are both safe and tumor-specific. Additional challenges involve the transient nature of epigenetic modifications and intratumoral heterogeneity, which restricts durability and clinical efficacy.

As DNMTi and HDACi continue to face limitations related to efficacy, toxicity, and resistance, targeted protein degradation (TPD) emerges as a complementary strategy capable of eliminating epigenetic regulators through proteasomal or lysosomal pathways. Proteolysis Targeting Chimera (PROTAC) and antibody-PROTAC (Ab-PROTAC) conjugates can selectively degrade key epigenetic proteins such as BET family members (e.g., BRD4), HDACs, EZH2, and others (Giardina et al., 2021). The review by Dai et al. (Dai et al., 2024) provides a comprehensive overview of TPD approaches, including PROTACs and alternative modalities such as molecular glues, ATTECs, SNIPERs, and LYTACs. These strategies have demonstrated superior antitumor activity compared with conventional inhibitors in leukemia, breast cancer, and xenograft models, largely through c-Myc downregulation and the ability to overcome resistance. Collectively, these advances highlight the potential of TPD to redefine epigenetic therapy by enabling complete removal of pathogenic proteins and guiding next-generation drug design toward improved selectivity and oral bioavailability.

Finally, biosynthetic nanoparticle platforms, including biomimetic nanoparticles, artificial exosomes, and co-delivery systems, have shown enhanced tumor specificity, improved drug retention, and markedly reduced systemic toxicity in animal models and early clinical studies. Mesenchymal stem cell-derived exosomes have been used to deliver therapeutic miRNAs such as miR-199a, increasing tumor sensitivity to chemotherapy while minimizing adverse effects (Lou et al., 2020). Nanoparticles cloaked with M1 macrophage-derived exosomal membranes have also improved intratumoral delivery of epigenetic inhibitors such as SAHA, resulting in selective tumor accumulation, stronger antitumor efficacy, and minimal systemic toxicity in lung cancer models (Li et al., 2021). Co-encapsulation strategies combining epigenetic drugs with chemotherapeutics in biomimetic nanoparticles further enhance tumor inhibition and reduce side effects, exemplified by the co-delivery of temozolomide with a bromodomain inhibitor in glioblastoma (Liu et al., 2022). Although most studies remain preclinical, advancing these systems into controlled clinical trials is essential, as they hold strong promise for improving efficacy and reducing cytotoxicity in epigenetic therapies.

## 7 Conclusion

Epigenetic regulation plays a pivotal role in the development and progression of cancer, as it can induce tumor suppressor gene silencing, activation of oncogenes, and genomic instability. In addition, several studies indicate that aberrant epigenetic profiles contribute to anticancer treatment resistance as reviewed elsewhere (Wang et al., 2023). Thus, targeting epigenetic regulators could provide additional strategies to overcome drug resistance and/or increase tumor sensitivity (Cano-Rodriguez et al., 2016; Nebbioso et al., 2012). While therapies targeting epigenetic regulators have achieved notable success and approvals by the regulatory agencies to treat hematologic malignancies (Table 1), their application in solid tumors is still in its early stages.

Taken together, the lack of specificity, high toxicity and poor bioavailability are usually pointed as the main reasons for treatment failure of solid tumors with epigenetic-based drugs. Additionally, the high complexity and interconnection of epigenetic mechanisms, as well as the high heterogeneity of epigenetic alterations in solid cancers (both inter- and intratumor) make it difficult to believe that targeting a single enzyme or a single mechanism might be the answer we are craving for. However, the combination of epigenetic drugs with other therapeutic approaches has shown promising results, especially in the era of immune therapy. Finally, the validation of epigenetic biomarkers for cancer diagnosis, prognosis and prediction of treatment response represents another poorly explored field with a high potential to improve cancer patient care.
