# Supplementary material for: The power and the promise of epigenetic drugs in oncology
Source: Front Genet. 2026 Jan 2;16:1622115. doi: 10.3389/fgene.2025.1622115 (PMC12807445; doi:10.3389/fgene.2025.1622115)
Supplement: Supplementary file 1 [file Table1.docx]

Supplementary Material

**Supplementary Table 1.** Clinical trials testing epigenetic drugs as monotherapy or in combination with other drugs for cancer treatment

| **NCT Number** | **Type of Cancer** | **Epi-Drug Intervention** | **Drug Class** | **As monotherapy or combinatory?** | **Phases** | **Enrollment** |
| --- | --- | --- | --- | --- | --- | --- |
| NCT00978250 | Head and Neck Cancer; Lung Cancer; Urinary Bladder Cancer; Breast Cancer | FdCyd | DNMTi | Combinatory | Phase 2 | 95 |
| NCT02085408 | AML | Decitabine | DNMTi | Combinatory | Phase 3 | 727 |
| NCT01534598 | Advanced Solid Tumors | FdCyd | DNMTi | Combinatory | Phase 1 | 59 |
| NCT01729845 | Relapsed/Refractory AML; MDS | Decitabine | DNMTi | Combinatory | Phase 1\| Phase 2 | 52 |
| NCT01861002 | Relapsed/Refractory Childhood ALL or AML | Azacitidine | DNMTi | Combinatory | Phase 1 | 15 |
| NCT01834248 | AML; CMML; MDS | Decitabine | DNMTi | Combinatory | Phase 1 | 9 |
| NCT01915498 | Advanced Hematologic Malignancies | Enasidenib | IDH2i | Monotherapy | Phase 1\| Phase 2 | 345 |
| NCT01928576 | Metastatic NSCLC | Azacitidine + Entinostat | DNMTi \| HDACi | Combinatory | Phase 2 | 101 |
| NCT01845805 | Pancreatic Cancer | Azacitidine | DNMTi | Monotherapy | Phase 2 | 49 |
| NCT02073994 | Advanced Solid Tumors; Glioma | Ivosidenib | IDH1i | Monotherapy | Phase 1 | 174 |
| NCT02273739 | Advanced Solid Tumors; Glioma; AITL | Enasidenib | IDH2i | Monotherapy | Phase 1\| Phase 2 | 21 |
| NCT02250326 | Advanced NSCLC | Azaciditine | DNMTi | Combinatory | Phase 2 | 240 |
| NCT02497404 | Myeloid Malignancies | Azacitidine | DNMTi | Combinatory | Phase 2 | 40 |
| NCT02518958 | Advanced Solid Tumors; Lymphoma | RRx-001 | HDACi \| DNMTi | Combinatory | Phase 1 | 12 |
| NCT02546986 | NSCLC | Azaciditine | DNMTi | Combinatory | Phase 2 | 100 |
| NCT02577406 | Advanced AML | Enasidenib | IDH2i | Monotherapy | Phase 3 | 319 |
| NCT02632708 | AML | Ivosidenib \| Enasidenib | IDH1i \| IDH2i | Combinatory | Phase 1 | 153 |
| NCT02512172 | Advanced Colorectal Cancer | Azacitidine \| Romidepsin | DNMTi \| HDACi | Combinatory | Phase 1 | 27 |
| NCT02677922 | AML; MDS; MPN | Ivosidenib \| Enasidenib + Azaciditine | IDH1i \| IDH2i \| DNMTi | Combinatory | Phase 1\| Phase 2 | 130 |
| NCT02813135 | Pediatric Recurrent/Refractory Malignancies | Enasidenib | IDH2i | Monotherapy | Phase 1\| Phase 2 | 460 |
| NCT02847000 | Metastatic Pancreatic Adenocarcinoma | Decitabine | DNMTi | Combinatory | Early phase 1 | 13 |
| NCT02989857 | Nonresectable/Metastatic Cholangiocarcinoma | Ivosidenib | IDH1i | Monotherapy | Phase 3 | 187 |
| NCT02846935 | Refractory/​Relapsed Lymphoid Malignancies | Decitabine | DNMTi | Combinatory | Early phase 1 | 7 |
| NCT02664181 | NSCLC | Decitabine | DNMTi | Combinatory | Phase 2 | 13 |
| NCT03164057 | AML | Azacitidine \| Decitabine | DNMTi | Combinatory | Phase 2 | 206 |
| NCT03173248 | AML | Ivosidenib + Azacitidine | IDH1i \| DNMTi | Combinatory | Phase 3 | 146 |
| NCT03263936 | Relapse/​Refractory AML | Decitabine + Vorinostat | DNMTi \| HDACi | Combinatory | Phase 1 | 37 |
| NCT03206021 | Recurrent/Refractory Pediatric Brain/Solid Tumors | Azacitidine | DNMTi | Combinatory | Phase 1 | 31 |
| NCT03220477 | Advanced NSCLC | Guadecitabine | DNMTi | Combinatory | Phase 1 | 28 |
| NCT03206047 | Recurrent Ovarian; Fallopian Tube; or Primary Peritoneal Cancer | Guadecitabine | DNMTi | Combinatory | Phase 1\| Phase 2 | 75 |
| NCT03179943 | Refractory/Resistant Urothelial Carcinoma | Guadecitabine | DNMTi | Combinatory | Phase 2 | 21 |
| NCT03383575 | MDS; AML; CMML | Azacitidine + Enasidenib \| Enasidenib | DNMTi \| IDH2i | Both | Phase 2 | 63 |
| NCT03417427 | AML | Decitabine | DNMTi | Combinatory | Phase 2 | 100 |
| NCT03445858 | Pediatric/Young Adults Relapsed/Refractory Solid Tumors; Lymphoma | Decitabine | DNMTi | Combinatory | Early phase 1 | 21 |
| NCT03471260 | Hematologic Malignancies | Azacitidine + Ivosidenib | DNMTi \| IDH1i | Combinatory | Phase1\|Phase2 | 96 |
| NCT03343197 | Glioma | Ivosidenib \| Vorasidenib | IDH1i \| IDH1/2i | Monotherapy | Phase 1 | 49 |
| NCT03498521 | Cancer of Unknown Primary Site | Ivosidenib | IDH1i | Monotherapy | Phase 2 | 790 |
| NCT03515512 | AML; CMML | Enasidenib | IDH2i | Monotherapy | Phase 1 | 23 |
| NCT03683433 | Recurrent/Refractory AML | Azacitidine + Enasidenib Mesylate | DNMTi \| IDH2i | Combinatory | Phase 2 | 50 |
| NCT03720366 | AML | Enasidenib | IDH2i | Monotherapy | Phase 1 | 40 |
| NCT03684811 | Advanced Solid Tumors; Glioma | Olutasidenib (FT-2102) \| FT-2102 + Azacitidine | IDH1i \| DNMTi | Both | Phase 1\| Phase 2 | 93 |
| NCT03366116 | Advanced Solid Tumors | Aza-TdC | DNMTi | Monotherapy | Phase 1 | 50 |
| NCT03719989 | Relapsed/Refractory DLBCL; Relapsed/Refractory NHL | Azacitidine | DNMTi | Combinatory | Phase 2 | 27 |
| NCT03812796 | Gastrintestinal Cancer | Domatinostat (4SC-202) | HDACi | Combinatory | Phase 2 | 75 |
| NCT03564821 | Myeloid Tumors | Ivosidenib | IDH1i | Monotherapy | Phase 1 | 18 |
| NCT03839771 | AML; MDS | Ivosidenib + Enasidenib | IDH1i \| IDH2i | Combinatory | Phase 3 | 968 |
| NCT03903458 | Advanced Melanoma | Tinostamustine | HDACi | Combinatory | Phase 1 | 21 |
| NCT03765229 | Advanced Melanoma | Entinostat | HDACi | Combinatory | Phase 2 | 11 |
| NCT03732703 | Relapsed/Refractory Multiple Myeloma | Enasidenib | IDH2i | Combinatory | Phase 1\| Phase 2 | 228 |
| NCT03744390 | MDS | Enasidenib | IDH2i | Monotherapy | Phase 2 | 68 |
| NCT03825796 | AML | Enasidenib Mesylate | IDH2i | Combinatory | Phase 2 | 2 |
| NCT03843528 | Myeloid Malignancies | Vorinostat + Azacitidine | HDACi \| DNMTi | Combinatory | Phase 1 | 15 |
| NCT03503409 | MDS | Ivosidenib | IDH1i | Monotherapy | Phase 2 | 68 |
| NCT04022005 | Relapsed/Refractory DLBCL | Chidamide | HDACi | Combinatory | Phase 2 | 54 |
| NCT03728335 | AML | Enasidenib Mesylate | IDH2i | Monotherapy | Phase 1 | 15 |
| NCT04049344 | Advanced Renal Cell Carcinoma | Decitabine | DNMTi | Combinatory | Phase 2 | 25 |
| NCT04176393 | Relapsed/Refractory AML | Ivosidenib | IDH1i | Monotherapy | Phase 1 | 30 |
| NCT04248595 | AML | Azacitidine | DNMTi | Combinatory | Phase 2 | 100 |
| NCT04075747 | AML | Enasidenib | IDH2i | Combinatory | Phase 1 | 57 |
| NCT04164901 | Residual/Recurrent Glioma | Vorasidenib | IDH1/2i | Monotherapy | Phase 3 | 331 |
| NCT04278781 | Advanced/Metastatic/Recurrent Chondrosarcoma | Ivosidenib | IDH1i | Monotherapy | Phase 2 | 17 |
| NCT04279379 | Relapsed/​Refractory/Advanced NK/​T-cell Lymphoma | Decitabine | DNMTi | Combinatory | Phase 2 | 20 |
| NCT03878524 | Advanced Solid Tumors; Hematological Malignancies | Enasidenib \| Panobinostat \| Vorinostat | IDH2i \| HDACi \| HDACi | Combinatory | Phase 1 | 2 |
| NCT04355858 | Luminal Advanced Breast Cancer | SHR2554 | EZH2i | Combinatory | Phase 2 | 319 |
| NCT04257448 | Advanced Pancreatic Cancer | Romidepsin \| Azacitidine \| Romidepsin + Azacitidine | HDACi \| DNMTi | Combinatory | Phase 1\| Phase 2 | 75 |
| NCT04414969 | NK/T-cell Lymphoma of Nasal Cavity | Chidamide | HDACi | Combinatory | Phase 2 | 35 |
| NCT04195555 | Advanced Solid Tumors; Lymphoma; Histiocytic Disorders | Ivosidenib | IDH1i | Monotherapy | Phase 2 | 49 |
| NCT04522895 | MDS; CMML; AML | Enasidenib | IDH2i | Monotherapy | Phase 2 | 50 |
| NCT04407741 | Advanced Solid Tumors; B-cell Lymphomas | SHR2554 | EZH2i | Combinatory | Phase 1\| Phase 2 | 100 |
| NCT04553393 | Refractory /Relapsed Aggressive B-NHL | Chidamide + Decitabine | HDACi \| DNMTi | Combinatory | Phase 1\| Phase 2 | 80 |
| NCT04611711 | PD-1 Resistant Digestive System Tumors | Decitabine | DNMTi | Combinatory | Phase 1\| Phase 2 | 60 |
| NCT04092179 | Relapsed/Refractory AML | Enasidenib | IDH2i | Combinatory | Phase 1\| Phase 2 | 27 |
| NCT04187703 | MDS; MDS/MPN Crossover Syndromes | Azacitidine + Decitabine | DNMTi | Combinatory | Early phase 1 | 20 |
| NCT04603001 | AML; MDS; CMML; MPNs | LY3410738 \| LY3410738 + Azacitidine | IDH1/2i \| DNMTi | Both | Phase 1 | 260 |
| NCT04250051 | Relapsed/Refractory AML | Ivosidenib | IDH1i | Combinatory | Phase 1 | 25 |
| NCT04493164 | AML; MDS | Ivosidenib | IDH1i | Combinatory | Phase 2 | 30 |
| NCT04281498 | MPN | Enasidenib | IDH2i | Combinatory | Phase 2 | 6 |
| NCT04088188 | Unresectable/Metastatic Cholangiocarcinoma | Ivosidenib | IDH1i | Combinatory | Phase 1 | 8 |
| NCT04831710 | Refractory/Relapsed Angioimmunoblastic T-cell Lymphoma | Chidamide | HDACi | Combinatory | Phase 2 | 83 |
| NCT04774393 | Relapsed/Refractory AML | ASTX727 + Ivosidenib \| ASTX727 + Enasidenib | DNMTi \| IDH1i \| IDH2i | Combinatory | Phase1\|Phase2 | 84 |
| NCT04705818 | Advanced Solid Tumors | Tazemetostat | EZH2i | Combinatory | Phase 2 | 173 |
| NCT05029141 | Relapsed/Refractory AML | Chidamide + Azacitidine | HDACi \| DNMTi | Combinatory | Phase 2 | 21 |
| NCT04056910 | Advanced Solid Tumors; Glioma | Ivosidenib | IDH1i | Combinatory | Phase 2 | 16 |
| NCT05010772 | AML | ASTX727 \| ASTX727 + Enasidenib \| ASTX727 + Ivosidenib | DNMTi \| IDH2i \| IDH1i | Both | Phase 1 | 125 |
| NCT04955938 | Myeloproliferative Neoplasms | Ivosidenib \| Enasidenib | IDH1i \| IDH2i | Combinatory | Phase 1 | 50 |
| NCT05282459 | MDS; CMML | Enasidenib Mesylate | IDH2i | Monotherapy | Phase 1\| Phase 2 | 48 |
| NCT03240211 | PTCL; CTCL | Decitabine | DNMTi | Combinatory | Phase 1 | 37 |
| NCT05178693 | Neuroendocrine Tumors | ASTX727 | DNMTi | Combinatory | Phase 1 | 27 |
| NCT05400993 | HR+/HER2- Breast Cancer | Chidamide | HDACi | Combinatory | Phase 2 | 59 |
| NCT05089370 | Mucosal Melanoma | Decitabine / Cedazuridine (DEC-C) | DNMTi | Combinatory | Phase 1\| Phase 2 | 30 |
| NCT04655391 | Relapsed AML | Decitabine \| Enasidenib Mesylate \| Ivosidenib | DNMTi \| IDH2i \| IDH1i | Combinatory | Phase 1 | 0 |
| NCT05209074 | Resectable Pancreatic Adenocarcinoma | Ivosidenib | IDH1i | Combinatory | Phase 1 | 16 |
| NCT05441514 | Relapsed/Refractory AML | Enasidenib Mesylate | IDH2i | Combinatory | Phase 1 | 15 |
| NCT05636514 | MDS; CMML; AML | ASTX727 | DNMTi | Combinatory | Phase 1 | 12 |
| NCT05484622 | Recurrent/Progressive Astrocytoma | Vorasidenib | IDH1/2i | Both | Phase 1 | 72 |
| NCT05896813 | PTCL | Chidamide | HDACi | Combinatory | NA | 30 |
| NCT05958719 | Nodal TFH Cell Lymphoma | Chidamide + Azacitidine | HDACi \| DNMTi | Combinatory | Phase 2 | 37 |
| NCT05317403 | Relapse/Refractory AML | Azacitadine + Vorinostat | DNMTi \| HDACi | Combinatory | Phase 1 | 40 |
| NCT05876754 | Advanced/Metastatic Cholangiocarcinoma | Ivosidenib | IDH1i | Monotherapy | Phase 3 | 220 |
| NCT05907057 | AML | Ivosidenib + Azacitidine | IDH1i \| DNMTi | Combinatory | Phase 3 | 245 |
| NCT05756777 | Relapsed/Refractory AML | Ivosidenib \| Enasidenib | IDH1i \| IDH2i | Combinatory | Phase 1 | 36 |
| NCT04203316 | Relapsed/Refractory AML | Enasidenib Mesylate | IDH2i | Monotherapy | Phase 2 | 10 |
| NCT05873244 | HCC | Zabadinostat (CXD101) | HDACi | Combinatory | Phase 2 | 44 |
| NCT05740449 | Relapsed ALL; Recurrent/Refractory Lymphoblastic Lymphoma | Decitabine | DNMTi | Combinatory | Phase 1\| Phase 2 | 26 |
| NCT06081829 | Nonresectable/Metastatic Cholangiocarcinoma | Ivosidenib | IDH1i | Monotherapy | Phase 2 | 10 |
| NCT05921760 | Nonresectable/Metastatic Cholangiocarcinoma | Ivosidenib | IDH1i | Combinatory | Phase 1\| Phase 2 | 92 |
| NCT06176989 | Malignant Sinonasal and Skull Base Tumors | Enasidenib | IDH2i | Monotherapy | Phase 2 | 30 |
| NCT06265545 | Recurrent/Refractory AML | Ivosidenib | IDH1i | Combinatory | NA | 120 |
| NCT06127407 | Advanced/Metastatic Conventional Chondrosarcoma | Ivosidenib | IDH1i | Monotherapy | Phase 3 | 136 |
| NCT06161974 | High-Grade Glioma | Olutasidenib | IDH1i | Combinatory | Phase 2 | 65 |
| NCT00359606 | Advanced Cancer | FdCyd | DNMTi | Combinatory | Phase 1 | 58 |
| NCT00005639 | Metastatic Solid Tumors | Azacitidine | DNMTi | Combinatory | Phase 1 | 34 |
| NCT00404326 | Cervical Cancer | Hydralazine/ Magnesium valproate | DNMTi/HDACi | Monotherapy | Phase 2 | 18 |
| NCT00404508 | Refractory Solid Tumors | Hydralazine/ Magnesium valproate | DNMTi/HDACi | Combinatory | Phase 2 | 15 |
| NCT00532818 | Metastatic Cervical Cancer | Hydralazine/ Magnesium valproate | DNMTi/HDACi | Monotherapy | Phase 3 | 143 |
| NCT00533299 | Ovarian Cancer | Hydralazine/ Magnesium valproate | DNMTi/HDACi | Monotherapy | Phase 3 | 211 |
| NCT00697879 | Advanced Solid Tumors | CHR-3996 | HDACi | Monotherapy | Phase 1 | 40 |
| NCT00776503 | MDS | Vorinostat | HDACi | Combinatory | Phase 1\| Phase 2 | 52 |
| NCT00715793 | Metastatic Melanoma | Decitabine | DNMTi | Combinatory | Phase 1\| Phase 2 | 39 |
| NCT01209520 | NSCLC | Azacitidine | DNMTi | Combinatory | NA | 6 |
| NCT01016990 | NHL; Hodgkin Lymphoma; CLL | Valproic Acid | HDACi | Monotherapy | Phase 2 | 52 |
| NCT01048034 | MDS; CMML | Azacitidine | DNMTi | Monotherapy | Phase 2 | 30 |
| NCT01120834 | Relapsed/Refractory DLBCL | Azacitidine + Vorinostat | DNMTi \| HDACi | Combinatory | Phase 1\| Phase 2 | 17 |
| NCT01301820 | AML | Azacitidine | DNMTi | Monotherapy | Phase 2 | 120 |
| NCT01829503 | AML | Decitabine | DNMTi | Combinatory | Phase 2 | 44 |
| NCT02074839 | Advanced Hematologic Malignancies | Ivosidenib | IDH1i | Monotherapy | Phase 1 | 291 |
| NCT02159820 | Ovarian Cancer | Decitabine | DNMTi | Monotherapy | Phase 2\|Phase 3 | 500 |
| NCT02489903 | Lung Cancer; Neuroendocrine Tumors; Ovarian Cancer | RRx-001 | HDACi/DNMTi | Combinatory | Phase 2 | 139 |
| NCT02499861 | Pediatric Relapsed/Refractory Malignancies | Decitabine | DNMTi | Combinatory | Phase 1\| Phase 2 | 6 |
| NCT02446652 | Cervical Cancer | Hydralazine/ Magnesium valproate | DNMTi/HDACi | Combinatory | Phase 3 | 230 |
| NCT02489929 | AML; MDS | Azaciditine | DNMTi | Monotherapy | NA | 35 |
| NCT02608437 | Metastatic Melanoma | Guadecitabine  (SGI-110) | DNMTi | Combinatory | Phase 1 | 19 |
| NCT02719574 | AML; MDS | Olutasidenib (FT-2102) \| FT-2102 + Azacitidine | IDH1i \| DNMTi | Both | Phase 1\| Phase 2 | 336 |
| NCT02705469 | mCRPC | ZEN003694 | BRDi | Monotherapy | Phase 1 | 44 |
| NCT03013998 | AML | Azacitidine\|Enasidenib + Azaciditine\|Ivosidenib + Azaciditine\|Decitabine | DNMTi \| IDH2i \|  IDH1i \| DNMTi | Combinatory | Phase1\|Phase2 | 2000 |
| NCT02711956 | mCRPC | ZEN003694 | BRDi | Combinatory | Phase 1\| Phase 2 | 75 |
| NCT05772728 | Relapsed/​Refractory nTFHL | Azacitidine | DNMTi | Combinatory | NA | 23 |
| NCT05615818 | Advanced Biliary Cancer | Ivosidenib | IDH1i | Combinatory | Phase 3 | 800 |
| NCT05609994 | Low-Grade Gliomas | Vorasidenib | IDH1/2i | Combinatory | Phase 1 | 48 |

Data regarding clinical trials were gathered at ClinicalTrial.Gov website (<https://clinicaltrials.gov/>, last accessed on February 22^nd^, 2024). The information in this table was gathered searching for terms “epigenetic therapy” and “cancer”, with the additional filtering: Recruitment status: Recruiting, Not yet recruiting, Active, not recruiting, Completed, Enrolling by invitation, Unknown status Studies; Eligibility Criteria: Sex: All; Study Type: Interventional (Clinical Trial). This search resulted in 145 studies that were further filtered to maintain only trials that were investigating an epigenetic-drug based monotherapy or in combination with other therapeutic agents in cancer (or other epi-drugs), resulting in 87 studies. Until the submission date of this review, all data obtained was reviewed and updated. However, it should be noted that several trials were not found when the filters above were applied. This suggests that additional trials may exist not captured by the search filters used here. Further data collection is required to explore why specific terms, such as “epigenetic therapy” does not include all trials involving epi-drugs. **Abbreviations**: AITL = Angioimmunoblastic T-cell lymphoma; ALL = Acute Lymphoblastic Leukemia; AML = Acute Myeloid Leukemia; CLL = Chronic Lymphocytic Leukemia; CMML = Chronic Myelomonocytic Leukemia; CTCL = Cutaneous T-cell Lymphoma; DLBCL = Diffuse Large B Cell Lymphoma; HCC = Hepatocellular carcinoma; mCRPC = Metastatic Castration-Resistant Prostate Cancer; MDS = Myelodysplastic Syndrome; MPN = Myeloproliferative Neoplasms; NHL= Non-Hodgkin Lymphoma; NSCLC = Non-Small Cell Lung Cancer; nTFHL = Intranodal follicular adjuvant T-cell lymphoma; PTCL = Peripheral T Cell Lymphoma; TFH = T-follicular Helper;
